# CuO Nanoparticles and Microaggregates: An Experimental and Computational Study of Structure and Electronic Properties

**DOI:** 10.3390/ma16134800

**Published:** 2023-07-03

**Authors:** Lorenzo Gontrani, Elvira Maria Bauer, Alessandro Talone, Mauro Missori, Patrizia Imperatori, Pietro Tagliatesta, Marilena Carbone

**Affiliations:** 1 Department of Chemical Science and Technologies, University of Rome “Tor Vergata”, Via della Ricerca Scientifica 1, 00133 Rome, Italy; lorenzo.gontrani@uniroma2.it (L.G.); atalone84@gmail.com (A.T.); pietro.tagliatesta@uniroma2.it (P.T.); 2 Italian National Research Council-Institute of Structure of Matter (CNR-ISM), Via Salaria km 29.3, 00015 Monterotondo, Italy; elvira.bauer@ism.cnr.it (E.M.B.); patrizia.imperatori@cnr.it (P.I.); 3 Institute of Complex Systems, National Research Council (CNR-ISC) and Department of Physics, Sapienza University of Rome, 00185 Rome, Italy; mauro.missori@isc.cnr.it

**Keywords:** nanoparticles, microaggregates, bandgap, DFT, diffuse reflectance spectroscopy, copper oxide, cluster models

## Abstract

The link between morphology and properties is well-established in the nanoparticle literature. In this report, we show that different approaches in the synthesis of copper oxide can lead to nanoparticles (NPs) of different size and morphology. The structure and properties of the synthesized NPs are investigated with powder X-ray diffraction, scanning electron microscopy (SEM), and diffuse reflectance spectroscopy (DRS). Through detailed SEM analyses, we were able to correlate the synthetic pathways with the particles’ shape and aggregation, pointing out that bare hydrothermal pathways yield mainly spheroidal dandelion-like aggregates, whereas, if surfactants are added, the growth of the nanostructures along a preferential direction is promoted. The effect of the morphology on the electronic properties was evaluated through DRS, which allowed us to obtain the electron bandgap in every system synthesized, and to find that the rearrangement of threaded particles into more compact structures leads to a reduction in the energy difference. The latter result was compared with Density Functional Theory (DFT) computational models of small centrosymmetric CuO clusters, cut from the tenorite crystal structure. The computed UV-Vis absorption spectra obtained from the clusters are in good agreement with experimental findings.

## 1. Introduction

The interest in nanomaterials has been increasing at a very steady pace in the last few years, as testified by the very large number of reports published in scientific and technological literature. The ‘aces up the sleeve’ of such systems, compared with other less coveted materials, lie in the vast range of technological properties they exhibit, that span from superconductivity to biosensing [1,2,3]. Among these materials, metal oxides have received much attention, owing to their role as chemical- and biosensors, (photo) catalysts [4,5,6,7,8,9,10], and photodetectors. Additional noteworthy properties of metal oxides include their antimicrobial power, with very limited release of harmful compounds as compared to traditional disinfection methods [11,12,13,14], which allows them to be used as a valid alternative in a variety of applications; most of the cited properties manifest themselves especially when the compounds are obtained in nanosized form [4,10,15,16,17,18,19,20,21,22,23,24,25].

Within the oxide pool, copper oxide (CuO), in particular, is finding several applications in devices, because of its p-type semiconductive nature. A noteworthy feature is the tunability of its bandgap, which can be either direct (2 eV–4 eV) or indirect (1 eV–1.4 eV) depending on the conditions (e. g. presence of surface and/or defect states, applied strain) and can be engineered by changing the latter [26,27,28]. Such a feature paves the way for its use in opto-electronic applications of different types and has recently been employed in gas sensing [29,30], magnetic storage [31], solar energy harvesting—transformation [32] and catalysis [33,34], as a photothermally active and photoconductive compound [35], as well as a biocide against several targets, including fungi [36,37,38], bacteria [39,40,41], or cellular lines (tumor [42] and macrophage [43]).

With regard to the morphological architectures of CuO nanoparticles (NPs), plate-, needle-, and wire-shaped arrangements and nano-cuboids, -platelets, -rods, and -belts with preferentially oriented crystal planes have been reported [44,45,46].

Regarding nanoparticle synthesis, two families of protocols have been developed so far, i.e., top-down and bottom-up methodologies, that are alternatively chosen depending on the starting reactants and on the desired target NP size/morphology. The latter process, often described as self-assembly, involves the aggregation of smaller fragments to form the desired structure, through different types of reactions, e.g., co-precipitation, sol–gel, hydrothermal–solvothermal, microemulsion, and chemical vapor [47,48,49,50,51,52,53,54]. In contrast, the top-down approach involves the etching or fragmentation of bulk precursors to form smaller structures, and includes mechanic actions like exfoliation, pulverization, grinding, milling, crushing, as well as laser ablation [55].

The attainment of nanosized particles with either method is an important requirement for the quantum confinement (QC) effect to operate [56], with consequent modulation of the bandgap and of the resulting opto-electronic properties; in addition, Buhro et al. [57] showed that the nanoparticle shape can have an effect on QC similar to that of the size. In particular, the bandgap of nano-sized CuO is often shifted from the bulk value, and ranges from 1.2 eV to 2.1 eV, according to Ogwu et al. [58]. Some of the reported shifts point towards larger energy values (blue), and bandgaps up to 3.02 eV in aligned nanoplatelets arrays [59] and 4.13 eV in 10-nm quantum dots [60] were described. Overall, the material is generally considered as a narrow-bandgap semiconductor [28,61,62]. The electronic structure of CuO was theoretically investigated using both cluster model calculations [63] and crystal phase band structure calculations, based on the local density approximation (LDA) [64] augmented with the on-site repulsive interaction potential U (LDA+U) [65] and on the local spin density approximation (LSDA+U) [66] to correctly take into account the electron correlation. Several many-body perturbation calculations of different sophistication levels were accomplished (e.g., [67,68,69]) by GW method (G = Green’s functions, W = Coulomb potential) which support a 1.2 eV value for the indirect gap of the bulk material.

Based on these premises, we performed a multi-technique study to further investigated the relation between the synthesis methods and the shape of the outcome CuO particles, and carried out an experimental assessment of the bandgap. The operative conditions of the bottom-up synthesis protocol were varied, and a thorough characterization of the product NPs and of the starting reactants by using several techniques was carried out. In particular, the synthesized materials were analyzed with (i) powder X-ray diffraction (P-XRD) in order to investigate the crystallinity and phase identification; (ii) infrared spectroscopy (IR) to assess purity and composition of both products (oxides) and reactants; (iii) scanning electron microscopy (SEM) to point out morphology and average dimensions of the NPs; and (iv) diffuse reflectance spectroscopy (DRS) to estimate the synthesized materials’ electronic/optical bandgap. The energy difference values were obtained from the Tauc plot extrapolation of the Kubelka–Munk function derived from DRS spectra. The values were compared with some new all-electron ab initio (DFT) calculations of the energy difference between HOMO (highest occupied molecular orbital) and LUMO (lowest unoccupied molecular orbital) energies, also known as electronic bandgap, and with TD-DFT calculations of the vertical absorption spectrum performed on cluster models, whose validity, in the form of both neutral and ionic systems, has been described in detail by several authors [70,71,72,73]. Notwithstanding the impossibility of performing calculations on model nanoparticles having dimensions comparable to the experimental ones (micrometric), as suggested by SEM images, CuO clusters of increasing complexity were taken into account, in order to derive a trend with respect to dimensions. Several “spherical” centrosymmetric aggregates of Cu and O atoms with 1:1 stoichiometry, cut from tenorite mineral monoclinic crystal [74,75], were essayed as small-scale replicas of the dandelion motifs observed in the SEM study. Therefore, the main aim of this study is to derive a multi-step structure–property relationship, starting from the first link between different preparative conditions and product morphologies, and proceeding through the second connection between morphologies and electronic properties. This approach may be valuable for the synthesis of task-specific nanoparticles, considering the marked variability in the technological properties of these systems.

## 2. Materials and Methods

### 2.1. Materials and Sample Preparation

Cu(NO_3_)_2_, NaHCO_3_, CO(NH_2_)_2_, citric acid and cetyltrimethylammonium bromide (CTAB) were purchased at Carlo Erba, Milan, Italy.

Five different approaches were used to synthesize CuO particles, three of which are procedures in autoclave, carried out either in a single step for a direct synthesis of CuO or in double steps, i.e., synthesizing a precursor that is afterwards calcined. The direct synthesis in autoclave was accomplished both with and without a templating agent (CTAB). In addition, synthesis with the sol-gel method, using citric acid and precipitation with NaHCO_3_, yielded the fourth and fifth CuO samples, respectively.

More in detail, in the two-step hydrothermal synthesis of CuO, the double salt copper hydroxycarbonate was achieved (Cu_2_(OH)_2_CO_3_ CuCO_3_·Cu(OH)_2_) in the first instance, through the reaction of copper nitrate (Cu(NO_3_)_2_), as a source of Cu^2+^ cations, with urea (CO(NH_2_)_2_). The latter molecule decomposes into ammonia and carbon dioxide at temperatures above 80 °C [76], thus gradually increasing the solution pH. CO_2_ and NH_3_ subsequently react in the alkaline solution with the copper ions present. The scheme below shows the reactions that can take place:

CO(NH_2_)_2_ + H_2_O → 2NH_3_ + CO_2_

CO_2_ + H_2_O → H_2_CO_3_ → CO_3_^2−^ + 2H^+^

NH_3_ + H_2_O → NH_4_^+^ + OH^−^

2Cu^2+^ + 2OH^−^ + CO_3_^2−^ → Cu_2_(CO_3_)(OH)_2_

The clear blue-colored solution prepared at the start (Cu(NO_3_)_2_ 0.006 M, urea 0.018 M, pH = 6.5) was heated at 100 °C for 6 h. At the end of the hydrothermal process, a pale green precipitate was produced in all preparations, and the pH of the supernatant liquid increased to 7. The solution was centrifuged, and the collected precipitate was subjected to calcination at 300 °C for 3 h following an initial ramp of 2 °C/min, to yield the sample labelled CuO-HT1.

The direct hydrothermal methods yielded CuO in the form of a black powder, without the need for subsequent calcination, after heating for 6 h at 180 °C. The base used for the precipitation step was NaHCO_3_, and the transition from the initial reactants to the final product took place in one step only (sample CuO-HT2). The 6 h hydrothermal synthesis at 180 °C, using urea as a base and CTAB as surfactant, also yielded a black powder (sample CuO-SF).

In the sol-gel synthesis of CuO, equimolar solutions of Cu(NO_3_)_2_ and citric acid were dissolved in distilled water, and the solution was heated up at 50 °C for 12 h, when a brilliant blue gel formed. The gel was subjected to quenching treatment, upon heating to 200 °C, yielding a porous grey solid that was subsequently grided and heated up to 500 °C, thus resulting in a black powder (Sample CuO-SG).

The synthesis through precipitation (non-hydrothermal) is carried out in two steps, with achievement of the precursor, using NaHCO_3_ as base and calcination of the precipitate.

In a typical synthesis, Cu(NO_3_)_2_ is dissolved in water and NaHCO_3_ is added as powder under magnetic stirring at room temperature, causing the formation of a light blue product. The vessel is sealed and kept under mechanical stirring (paddle mill) for 3 h, to allow digestion in the presence of CO_2_ according to the overall reactions:

HCO_3_^−^ → CO_3_^2−^ + H^+^

HCO_3_^−^ + H_2_O → OH^−^ + H_2_CO_3_

H_2_CO_3_ → CO_2_ + H_2_O → CO_3_^2−^ + 2H^+^

2Cu^2+^ + 2OH^−^ + CO_3_^2−^ → Cu_2_(CO_3_)(OH)_2_

**Scheme 1:**
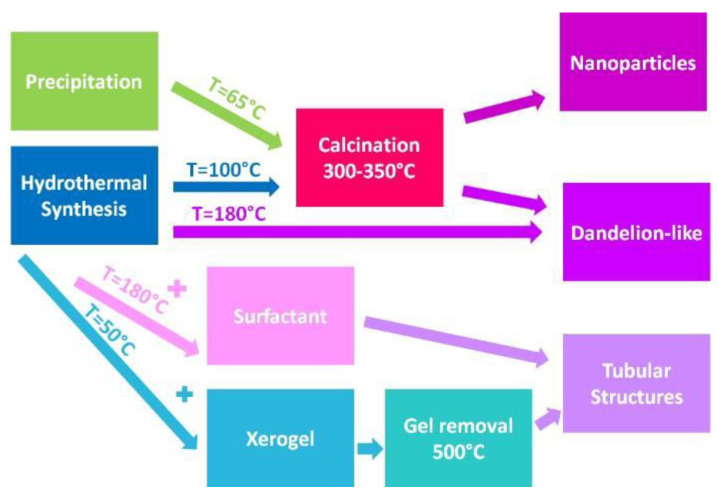
A systematic diagram of the conditions used for the syntheses of the CuO microaggregates and nanoparticles.

The pale green to light blue precipitate is recovered by filtration, washed with distilled water, dried at 80 °C, and calcined at 350 °C for 3 h to yield a black powder (sample CuO-Prec). A summary of the synthesis conditions is reported in Table 1. A systematic diagram of the conditions used for the syntheses, as well as the type of particles that were achieved, is reported in Scheme 1.

### 2.2. Apparatuses

The powder diffraction patterns were obtained with a Seifert 3003TT diffractometer (Malvern Panalytical, Almelo, The Netherlands). Scans were taken with a 2θ step size of 0.02° with counting time 2 s/step, using Cu Ka radiation.

Infrared spectra were recorded with a Shimadzu Prestige-21 FT-IR instrument (Kyoto, Japan), equipped with an attenuated total reflectance (ATR) diamond crystal (Kenmore, WA, Kyoto, Tokyo, Japan), in the range 400–4000 cm^−1^, with a resolution of 4 cm^−1^.

The surface morphology of the oxides was determined with Zeiss Auriga field emission–scanning electron microscope (SEM, Oberkochen, Germany) operating at 6–8 kV. The EDS maps were taken by coupling the field emission–scanning electron microscope (SUPRA™ 35, Carl Zeiss SMT, Oberkochen, Germany) with energy dispersive microanalysis (EDS/EDX, INCAx-sight, Model: 7426, Oxford Instruments, Abingdon, Oxfordshire, UK), operating at 20 KV. Samples were deposited on silicon sample-holders by drop-casting of ethanol suspensions, achieved by mild sonication. Open-source ImageJ software was used for image analysis. Optical measurements in the ultraviolet (UV), visible (Vis), and near infrared (NIR) spectral regions were obtained by a diffuse reflectance setup from Avantes BV (Apeldoorn, The Netherlands). The latter comprises a combined deuterium–halogen radiation source (AvaLight-DH-S-BAL-Labsphere, Lafayette, CO, USA) connected via an optical fiber to a 30 mm diameter Spectralon^®^ coated integrating sphere (AvaSphere-30-REFL-Labsphere, USA), used to illuminate the samples (sampling port diameter 6 mm) and to collect the radiation diffusely reflected. The samples used for DRS measurements were prepared from each synthesized nanoparticle batch (see Section 2.1) by mixing 20 mg of CuO with 480 mg of the highly reflective compound BaSO_4_. The dilution brought about by this mixing ensures that the resulting powder has low absorption. The compounds were placed in the well of a sample holder with a depth such as to be able to consider the reflectance spectra as those of an infinitely thick sample ($R_{\infty }$). The integrating sphere was connected through another optical fiber to a spectrometer (AvaSpec-2048 × 14-USB2). This configuration allows applications in the 248–1050 nm range with a 2.4 nm spectral resolution. A laptop was used for the spectrometer control and data recording, whereas a factory-calibrated Spectralon^®^ (Labsphere, North Sutton, NH, USA) was used as the reflectance reference. Each reflectance spectrum was obtained by averaging five acquisitions lasting 5 s each.

### 2.3. Computations

The computational study was carried out with density functional theory methods. Four hybrid density functionals among those available in the Gaussian software (Gaussian 16 Rev. A.03) were tried, namely PBE0 [77], HSE06 [78], and the long-range-corrected CAM-B3LYP [79] and LCwHPBE [80]. The spherical clusters subject to the calculations were “dug” into the tenorite monoclinic crystal structure taken from the American Mineralogist Database [74,75] by generating a 2 × 2 × 2 structure from the unit cell with the software Mercury 2021.3.0 [81], followed by the identification of the center of geometry with an in-house code and of the atoms contained in spheres of given radii centered on that point with VMD tools [82]. Two types of models were investigated: in the first batch, the geometry of the system was kept fixed at such “experimental” values throughout the entire calculation. To ensure proper convergence in this case, the system wavefunction was progressively refined along a series of calculations, i.e., following the order PME6 (semiempirical)->HF/6-31G(d)->final DFT/6-31 + G(d), and using the optimized wavefunction of each cycle as a starting guess for the next one. The SCF protocol was tuned, by switching from the default to the quadratically convergent SCF method. A satisfactory final convergence, smaller than 10^−8^ atomic units and consequently appropriate for higher-level calculations (gradient, perturbative, etc.), was obtained for the models containing 8, 16, 24, 38, and 44 Cu-O pairs only using the LCwHPBE functional, whereas the maximum convergence obtained with 58 atom pairs was 10^−5^ only. The energy calculation of the first five performing models was followed by a linear response TD-DFT expansion with a number of states (up to 65) progressively increasing with the cluster dimension, in order to cover approximately the same wavelength range across all models. A more complete study was performed afterwards for the three smallest systems (8, 16, 24 Cu-O pairs), by optimizing the structure of the cluster in vacuo with gradient methods, until the threshold of 4.5 × 10^−4^ Hartree/Bohr was reached. The nature of the stationary point found was checked by performing a force constant/frequency calculation, and in any case, no imaginary frequencies were obtained, confirming that the structure was actually a local minimum on the potential energy surface. All the calculations were performed with the Gaussian 16 program [83] and visualized with Molden (Molden 5.9) [84] and Gaussview (GV 6) [85] software. The latter program was used to draw the theoretical spectra by convolution of the calculated vertical transitions, considering a 0.05 eV half width at half maximum (HWHM) for each line.

## 3. Results and Discussion

### 3.1. Characterization of Precursors

#### 3.1.1. FTIR Spectra

**Figure 1:**
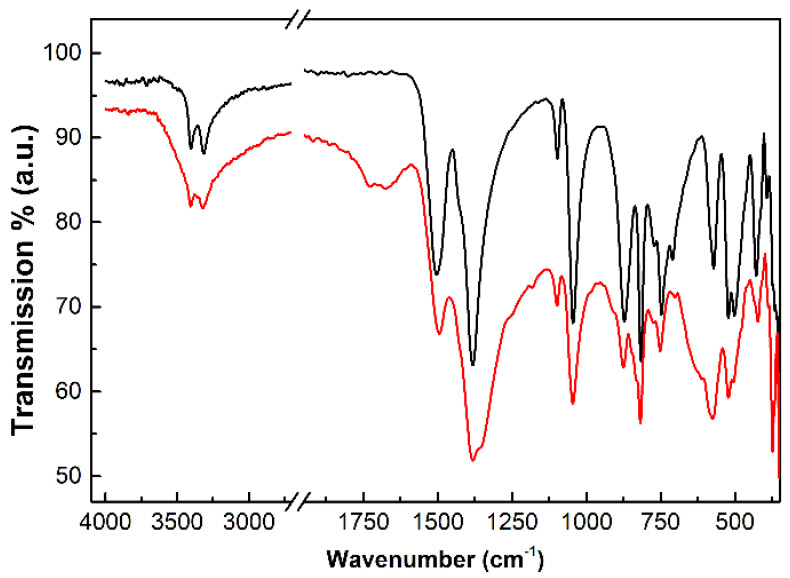
Experimental FT-IR (ATR) spectra of the precursors (copper hydroxide carbonate) of CuO-HT1 (black line) and CuO-prec (red line).

**Figure 2:**
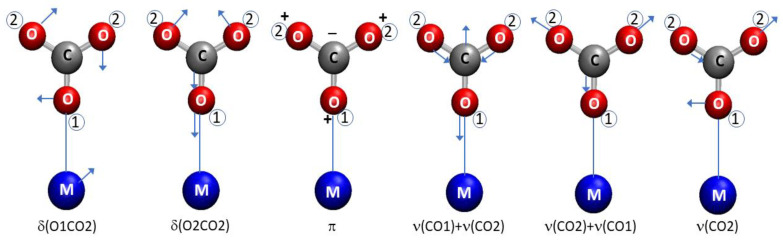
Typical normal modes of metal carbonate complexes. The oxygen atoms of the carbonate are labelled as ① if bonded, and ② if not-bonded to the metal.

The mid-infrared transmission spectra (400–4000 cm^−1^) of the hydroxycarbonate precursor of CuO-HT1 and CuO-prec is reported in Figure 1. The fingerprint region is very similar between the two, and the following principal features can be pointed out, according to the carbonate anion normal mode analysis reported in Figure 2 below, reproduced from [85,86]:

- 3404 and 3313 cm^−1^, OH stretching, redshifted due to the interaction with the metal cation;
- 1504 and 1381 cm^−1^, coordinated and non-coordinated C-O stretching;
- 1095, 1045, 873 cm^−1^, O-H bending and C-O + O-Cu coupled stretching;
- 815 and 748 cm^−1^, out-of-plane bending and antisymmetric O-C-O group vibration.
- The peaks falling below 600 cm^−1^ are related to Cu-O skeleton vibration.

**Figure 3:**
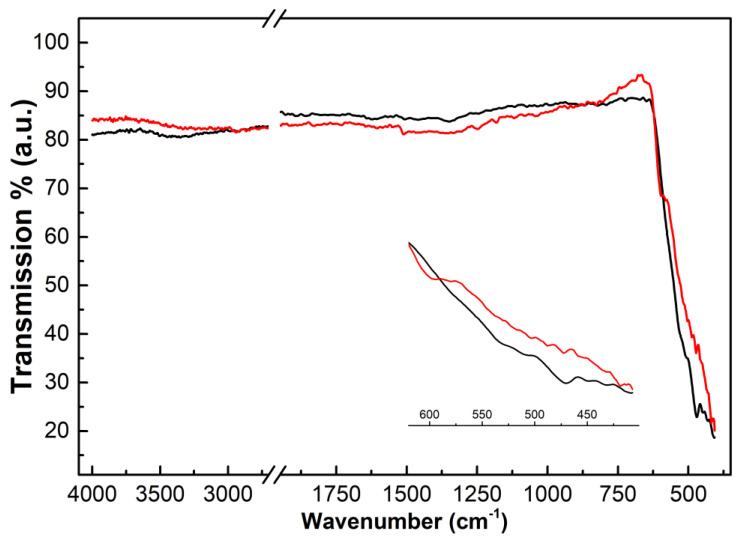
Experimental FT-IR (ATR) spectra of the final copper oxide products. CuO-HT1 (black line) and CuO-prec (red line). Inset: magnification of 400–620 region.

As for the final products, Figure 3 shows the ATR infrared spectra of CuO-HT1 and CuO-Prec. The measured spectra are rather featureless in the 700–4000 cm^−1^ region, whereas a multibranch absorption peak is evident in the lower frequency region in the range 400–700 cm^−1^. The peak appears to have a fine structure, with absorptions around 420, 460, and 610 cm^−1^. Though studies on infrared and Raman spectra of CuO are not very frequent in literature, the presence of such peaks was evidenced in a 1991 study on single crystals and was attributed to one A_u_ and two B_u_ modes [87,88]. More recently, investigations devoted to CuO nanoparticles pointed out absorptions at 436, 504, and 610 cm^−1^ [89], and at 430, 490, and 615 cm^−1^ [90], respectively, whereas the triplet occurs at different values in the range 428–608 cm^−1^, depending on the specific nanoparticle [91]. The peaks were attributed to Cu-O stretching vibrations occurring in different lattice directions. In our case, very similar patterns are found for all synthesized CuO samples.

#### 3.1.2. XRD

**Figure 4:**
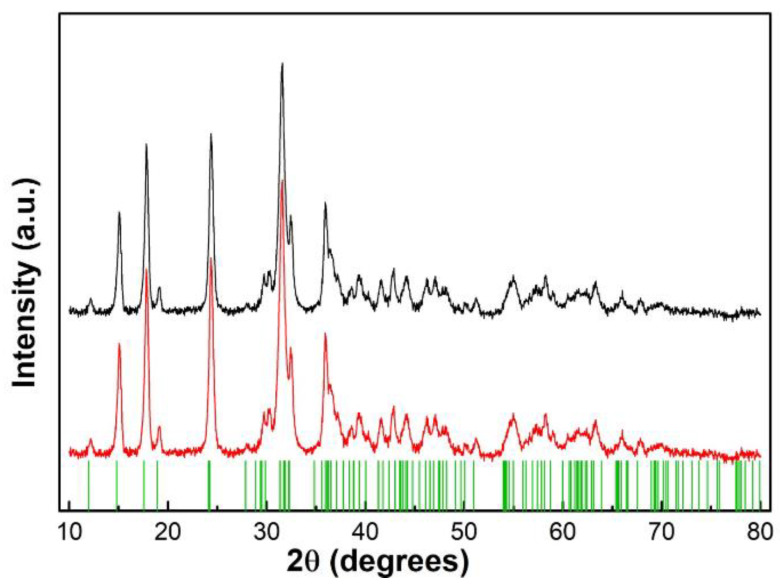
X-ray powder diffraction patterns of CuO precursors. Black: CuO-HT1; Red: Cuo-Prec. Green vertical lines: malachite mineral Bragg X-ray reflections.

**Figure 5:**
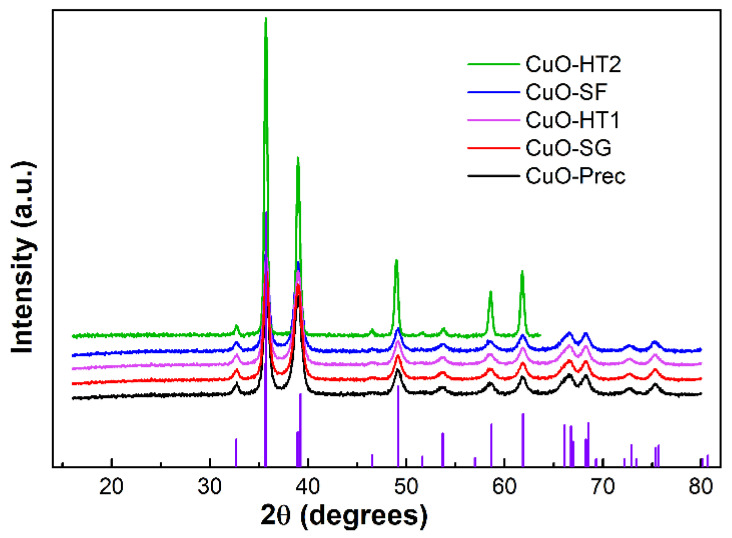
Powder X-ray diffraction patterns of the synthesized CuO samples. Bottom area vertical lines: tenorite mineral Bragg X-ray reflections.

The powder X-ray diffraction patterns of the precursor system employed as reaction intermediate for CuO-HT1 and CuO-Prec syntheses (copper hydroxide carbonate) are reported in Figure 4. The two profiles are superimposable, signaling two identical structures. The curves were compared with the Bragg reflections of the malachite mineral crystal, having the same Cu_2_(CO_3_)(OH)_2_ composition [92], and optimal correspondence was found. An XRD analysis of all final products obtained was performed as well, with the aim of investigating their solid structure. The measured diffraction profiles of the five samples (see Table 1, first column, for the sample identification) are reported overlaid in Figure 5 together with the X-ray reflections of tenorite crystal, the mineral where the Cu(II) oxide occurs in nature [93], which are shown as vertical lines at the bottom of the figure. By comparison among the diffraction profiles, considering both peak positions and intensities, it could be verified that all samples contained cupric oxide at high purity, confirming the predictions made from the analysis of infrared spectra.

#### 3.1.3. SEM Analysis

**Figure 6:**
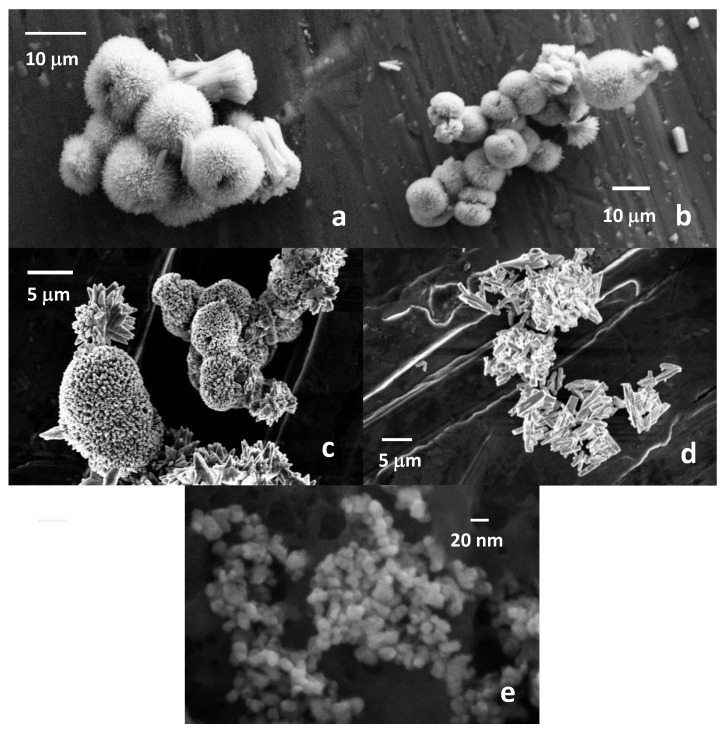
SEM images of the synthesized CuO nanoparticles: (**a**) CuO-HT1, magnification 2K; (**b**) CuO-HT2, magnification 2K; (**c**) CuO-SF, magnification 5K; (**d**) CuO-SG, magnification 2K; (**e**) CuO-Prec, magnification 800K.

In addition to XRD analysis, the synthesized samples were evaluated by SEM in order to get an insight in their morphology. Typical images were taken upon the drop casting of sample suspensions on a silicon sample holder and are reported in Figure 6. Samples synthesized by the hydrothermal method, in the absence of surfactants, appear to be composed of microaggregates of spheroidal filaments in a dandelion-like arrangement, whose diameter was estimated between 20 and 100 nm (CuO-HT1, Figure 6a), with the aid of an open-source software for imaging analysis (ImageJ). These microaggregates tend to re-arrange into a larger structure (CuO-HT2, Figure 6b). The closed dandelion surface is fringed into a duster-like particle in some cases, a feature that is observed more clearly in CuO-HT2 samples (Figure 6b). Dandelion-like structures may be observed in “high-temperature” hydrothermal synthesis (i.e., at temperatures ≥180°) [94] as well as in two-step synthesis, i.e., “low temperature” hydrothermal synthesis followed by calcination [95] and can be considered the outcome of nucleation and growing processes shaped by an interplay of pressure and temperature, which causes aggregation, anisotropic growth, and consequent elongation in a preferential direction, followed by aggregation into microstructures. In addition, the calcination step plays a role in the achievement of more highly ordered dandelion structure, by thermal rearrangement (Figure 6a).

The use of a long (n-hexadecyl) alkyl chain surfactant, such as CTAB, as well as the gel-like network typically formed in the sol-gel procedure offers an alternative growth pathway to the nanoparticles, that induces a partial tubular extension of the nanostructure, as can be seen in Figure 6c and, particularly, in Figure 6d. The more compact structures are definitively the outcome of the 3D frame created either by the surfactant or by the xerogel, during the synthetic process that confines the growth in the hollow spaces of the frame. In addition, the xerogel is removed at a higher temperature as compared to the calcination (500 °C), resulting in larger structures, with an average diameter of the short dimension of 200–700 nm. Tubules of CuO-SF have, for a large part, a diameter of 100–200 nm and are re-arranged in a globular structure. When a synthetic precipitation procedure is followed, including precipitate digestion (Figure 6e), nanoparticles are achieved with size in the range 10–20 nm.

#### 3.1.4. Diffuse Reflectance Spectroscopy

The opto-electronic behavior of all synthesized oxide nanoparticles was investigated by DRS, a technique that has proven to yield good estimates of the electronic bandgap of a large variety of materials. The bandgap of the CuO nanoparticle samples was estimated by measuring $R_{\infty }.$ This pattern, which corresponds to the reflectance of an infinitely thick layer of the sample, can be converted into absorption spectra according to the Kubelka–Munk function (K-M) [96,97]:

$$\frac{K}{S}=\frac{{\left(1-R_{\infty }\right)}^{2}}{2R_{\infty }}$$

where *K* and *S* are, respectively, K-M absorption and scattering coefficients. The former quantity is related to the intrinsic absorption coefficient of the particles α by α = *K*/2, in the case of diffuse light distribution, as in our case [98]. For a batch of different preparations of the same material (here CuO) with a similar method for preparing the solid mixture needed for the optical measurements, *S* can be considered constant from sample to sample, giving the ratio *K/S* ≈ α. In order to estimate the bandgap from the absorption spectra, the Tauc relation [99] was used:

αhν = C1(hν − E_g_)^*n*^

**Figure 7:**
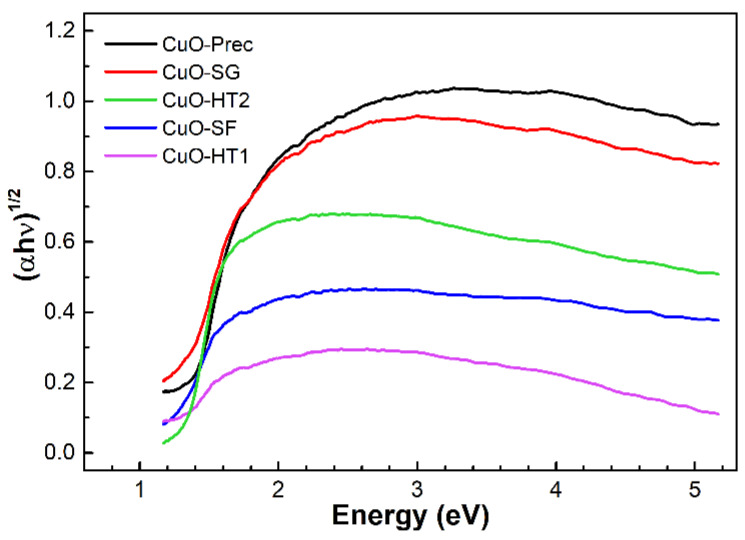
Diffuse reflectance spectra of the synthesized CuO nanoparticles. Color legend in the inset.

In this formula, C1 is a constant, whereas the exponent *n* = 2 and *n* = 1/2 apply to direct and indirect gaps (as in the present investigations), respectively. The patterns are reported in Figure 7 for all samples, whereas the fitted bandgap values are reported in Table 2. The bandgap values comply well with literature data and appear to correlate (apart from sample CuO-prec) with the preparation strategy and with the corresponding morphology (see Section 3.1.3). The particles obtained with the hydrothermal synthesis plus calcination show bandgaps smaller by 0.15–0.2 eV as compared to those synthesized without the final calcination step (CuO-HT2), suggesting that the presence of the dandelion superstructure into which the NP filaments rearrange at higher temperature lowers the energy difference.

#### 3.1.5. Theoretical Spectra

**Figure 8:**
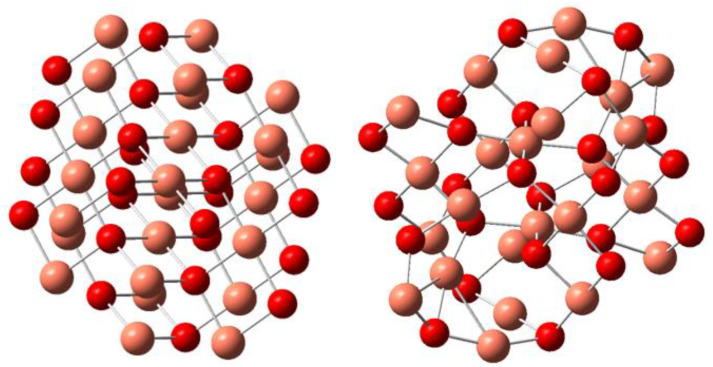
CuO24 model geometry. (**Left**): crystal model; (**Right**): optimized structure. Color legend: Red: oxygen atoms; pink; copper atoms.

A theoretical study was carried out on “spherical” centrosymmetric models cut from the crystal structure of tenorite crystal, containing 8, 16, 24, 38, and 44 CuO pairs. In the smallest aggregates (8, 16, 24 ion pairs), the geometry of the system was relaxed, by energy minimization. A picture of the two 24-member models is shown in Figure 8, whereas pictures of all the models are reported in the supplementary information, together with the coordinates of every aggregate (Figures S1–S8). 

**Figure 9:**
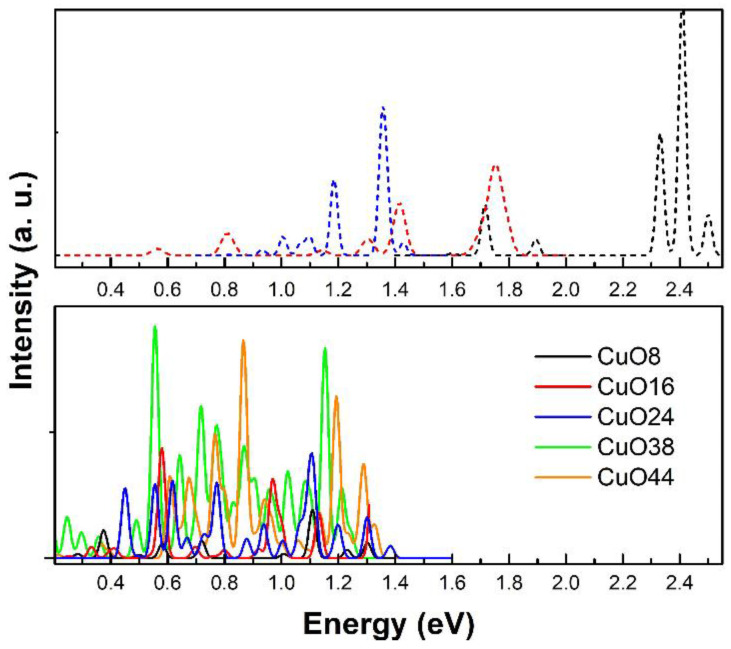
TD-DFT Calculated UV-VIS absorption spectra of the five “spherical” models in the range 0–2.6 eV. **Upper** panel: Relaxed clusters; **Lower** panel: Rigid clusters (see text). Color codes: black CuO8, red: CuO16; blue: CuO24; green: CuO38; orange: CuO44. Dashed lines: relaxed models; coninous lines: rigid models.

The optimization process that maintained the original C_i_ point symmetry resulted in a slight elongation of the aggregates as a consequence of the shortening of first-neighbor Cu-O bonds, though only a few defects were introduced, and the [4,4] Cu-O coordination was fairly wellmaintained. Regarding the bandgap calculations, two different types of bandgaps can be obtained from these models: the ground state calculation directly yields the energy difference between HOMO and LUMO molecular orbitals (“electronic bandgap”, reported in Table 3), whereas the TD-DFT calculation gives the vertical absorption spectrum and correspondingly hints at the “optical bandgap”, i.e., the energy threshold at which photons are absorbed in semiconductors, which is generally lower than the HOMO-LUMO gap, owing to excitonic effects in the excited state [100]. Concerning the former type of bandgap, the calculated values follow a monotonic decreasing trend with the number of atoms, except the lower outlier value found for (CuO)_38_. This type of gap reduction is very common in nanomaterials, and in conjugated pi-systems, it has a simple analogue in the ‘Particle in a Box’ model and an opposite in the quantum confinement effect [101]. Regarding modeling the excited states through TD-DFT, a variable number of vertical transitions was considered in the calculations, increasing by the system dimension, and was chosen sufficiently large in order to reach at least 1.4 eV, i.e., larger than the experimental estimated values by more than 0.1 eV. In particular, 20 states were employed for 8 CuO pairs, 40 for 16, and 24 and 65 for 38 and 44 pairs, respectively. It should be noted that the calculation of the bandgap depends largely on the type of functional considered, as already documented, e.g., by some of use for organic dyes [102]; in the calculations considered for this material, that were performed with the LCwHPBE functional that ensured proper SCF convergence in all models, the lowest energy wavelength found is always redshifted with respect to the experimental K-M gaps. The spectra of the two families of models (rigid and relaxed) are reported in Figure 9, with relaxed models in the top panel and rigid aggregates in the lower one. The most evident effect of the geometry optimization is the general blue shift linked to the shrinkage of the structures and the resulting quantum confinement effect discussed above, accompanied by a larger dispersion in energy (Figure 9, upper panel). Unfortunately, the calculated TD-DFT transitions (and excited states) contain contributions of a large number of ground state orbitals and therefore cannot be assigned easily. Additionally, the 24-pair optimized model gives origin to two intense peaks around 1.2 and 1.4 eV, ascribable to a transition with sizable HOMO–LUMO character and oscillator strength. This result complies well with the fitted experimental values obtained for “dandelion” samples and suggests that the reduced scale models can interpret the phenomenon, at least fairly.

The optimized models were also employed to calculate the infrared vibrational spectrum that is reported in the additional material (Figure S9). The patterns obtained show a larger number of far-infrared bands in the larger models, and can account, at least qualitatively, for the presence of the fine structure peaks observed in the experimental pattern between 400 and 600 cm^−1^. The bands below 500 cm^−1^ are predicted to be mainly contributed by bending modes, whereas the more intense absorption signals around 650 cm^−1^ are mainly stretching vibrations. It should be considered that the theoretical infrared spectrum has peaks falling in the range 30–798 cm^−1^, thus positioning the intense C-O stretching normal mode vibrations in the highest frequency range, whereas in the experimental profile (4000–400 cm^−1^), the same vibration occurs at the lowest energy side; more extended models and far-infrared spectroscopy experiments would be necessary to investigate this issue further.

## 4. Conclusions

In the present contribution, we synthesized CuO in different morphologies ranging from microaggregates to nanoparticles, following five different synthesis pathways. Synthetic strategies were based on hydrothermal, sol-gel, and precipitation strategies, including both direct CuO synthesis and CuO synthesis via a precursor. The vibrational properties and the powder X-ray diffraction patterns of precursors and final CuO products were assessed, indicating the formation of hydroxycarbonates, with a malachite crystalline structure, for the former and tenorite for the latter. The various morphologies and average dimensions of the CuO samples were investigated by SEM. The microscopy study showed that the samples prepared according to hydrothermal strategies are composed of filaments with a diameter in the 20–100 nm range, that tend to aggregate into dandelion structures, fringed into a duster-like arrangement, upon calcination. When a surfactant is used or the sol-gel method is applied, longer tubular motifs, with a compact structure, can be obtained, with a size of the shortest dimension ranging between 100 and 200 nm in the former case and between 200 and 700 nm in the latter case.

The electronic properties of all the NPs were finally appraised with DRS spectroscopy, and the observed bandgap values were related to the differences in nanoparticle morphology. The electronic properties of dandelion structures were also successfully modeled with DFT calculations, employing some centrosymmetric atomic models generated from the crystal structure of mineral tenorite (cupric oxide). The calculated UV-Vis absorption spectra, based on centrosymmetric CuO clusters, are in good agreement with experimental findings.

## Figures and Tables

**Table 1:** Details of synthesis pathways explored in the study.

Sample	[Cu(NO_3_)_2_]	Method	Base/Gel	Ratio	Surfact.	Temp.	Time	pH	Precursor	Calcination
CuO-HT1	0.1 M	Hydroth.	CO(NH_2_)_2_	1:3	-	100 °C	6 h	6.5–7	Cu_2_(CO_3_)(OH)_2_	300 °C–3 h
CuO-HT2	0.1 M	Hydroth.	NaHCO_3_	1:3	-	180 °C	6 h	-	No	NO
CuO-SF	0.06 M	Hydroth.	CO(NH_2_)_2_	1:3	CTAB	180 °C	6 h	-	No	NO
CuO-SG	0.04 M	Sol-Gel	Citric Acid	1:1	-	50 °C	12 h	-	No	500 °C *
CuO-Prec	0.07 M	Precip.	NaHCO_3_	1:5	-	65 °C	3 h	10–8.5	Cu_2_(CO_3_)(OH)_2_	350 °C–3 h

* temperature of gel breaking.

**Table 2:** Experimental bandgaps from Tauc plot extrapolation of Kubelka–Munk functions obtained from the DRS $R_{\infty }.$ (See text) and diameter range of the various structures.

Sample	Gap (eV)	Particle Size (nm)
CuO-HT1	1.10	20–100 *
CuO-HT2	1.30	20–100 *
CuO-SF	1.16	100–200 *
CuO-SG	1.18	200–700 *
CuO-Prec	1.26	10–20 nm

* diameter of the shortest dimension of the anisotropic dandelion and tubule structures.

**Table 3:** LCwHPBE HOMO–LUMO gaps for the tenorite “spherical” models.

	E(HOMO) a.u	E(LUMO) a.u	E(HOMO) eV	E(LUMO) eV	Gap eV
CuO8	−0.29244	−0.13219	−7.96	−3.60	4.36
CuO16	−0.30306	−0.14384	−8.25	−3.91	4.33
CuO24	−0.31875	−0.16348	−8.67	−4.45	4.23
CuO38	−0.27582	−0.17094	−7.51	−4.65	2.85
CuO44	−0.30197	−0.15239	−8.22	−4.15	4.07

## Data Availability

Data will be made available by the authors upon request.

## Supplementary Materials

The following supporting information can be downloaded at: https://www.mdpi.com/article/10.3390/ma16134800/s1, Figures S1–S5: Atomic coordinates of rigid cluster models (Figure S1: CuO8, Figure S2: CuO16, Figure S3: CuO24, Figure S4: CuO38, Figure S5: CuO44); Figures S6–S8: Atomic coordinates of relaxed cluster models: Figure S6: CuO8; Figure S7: CuO16; Figure S8: CuO24; Figure S9: Theoretical infrared spectrum calculated with DFT for CuO8 and CuO16 relaxed models.
