# Supplementary material for: CuO Nanoparticles and Microaggregates: An Experimental and Computational Study of Structure and Electronic Properties
Source: Materials (Basel). 2023 Jul 3;16(13):4800. doi: 10.3390/ma16134800 (PMC10343626; doi:10.3390/ma16134800)

CuO Nanoparticles and Microaggregates: An Experimental and Computational Study of Structure and Electronic Properties

Supplementary Information

1) Atomic coordinates of the tenorite cluster models

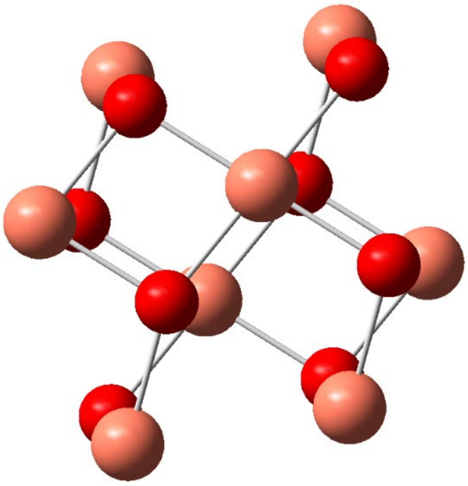

CuO8

Figure S1. CuO8.

16

O	-2.559000	-0.279000	1.263000
O	-2.121000	0.279000	-1.263000
Cu	1.607000	-0.855000	-2.525000
Cu	1.170000	-2.566000	0.000000
O	-0.219000	-1.989000	1.263000
Cu	-1.170000	-0.855000	0.000000
Cu	0.732000	-0.855000	2.525000
O	0.219000	-1.431000	-1.263000
O	2.121000	-0.279000	1.263000
Cu	-0.732000	0.855000	-2.525000
Cu	1.170000	0.855000	0.000000
O	-0.219000	1.431000	1.263000
Cu	-1.607000	0.855000	2.525000
Cu	-1.170000	2.566000	0.000000
O	0.219000	1.989000	-1.263000
O	2.559000	0.279000	-1.263000

CuO16

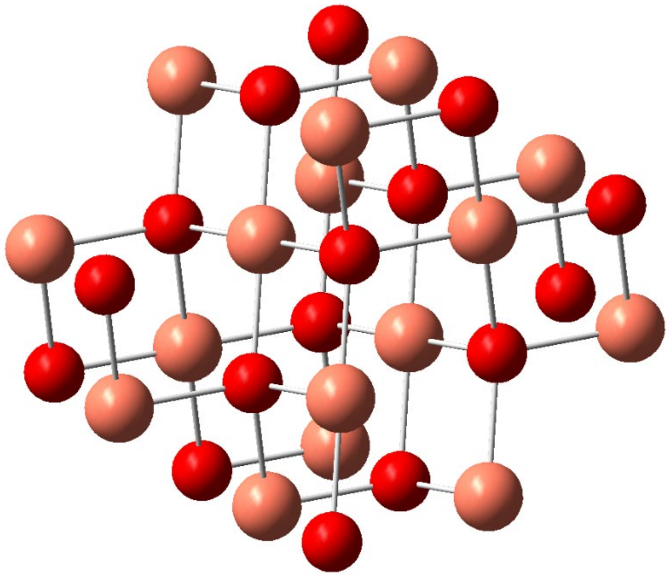

Figure S2.

CuO16.

32

Cu	-3.072000	-0.855000	-2.525000
O	-1.683000	-0.279000	-3.787000
O	-2.559000	-0.279000	1.263000
O	-2.121000	-3.141000	-1.263000
Cu	-3.510000	0.855000	0.000000
O	-2.559000	3.141000	1.263000
O	-2.121000	0.279000	-1.263000
Cu	-0.732000	-2.566000	-2.525000
Cu	1.607000	-0.855000	-2.525000
Cu	1.170000	-2.566000	0.000000
O	-0.219000	-1.989000	1.263000
Cu	-1.607000	-2.566000	2.525000
Cu	-1.170000	-0.855000	0.000000
Cu	0.732000	-0.855000	2.525000
O	0.219000	-1.431000	-1.263000
O	2.121000	-0.279000	1.263000
O	2.559000	-3.141000	-1.263000
O	0.656000	1.431000	-3.787000
Cu	-0.732000	0.855000	-2.525000
Cu	1.607000	2.566000	-2.525000
Cu	1.170000	0.855000	0.000000
O	-0.219000	1.431000	1.263000
Cu	-1.607000	0.855000	2.525000
Cu	-1.170000	2.566000	0.000000
Cu	0.732000	2.566000	2.525000
O	0.219000	1.989000	-1.263000
O	2.121000	3.141000	1.263000
O	2.559000	0.279000	-1.263000
O	-0.656000	-1.431000	3.787000
Cu	3.510000	-0.855000	0.000000
Cu	3.072000	0.855000	2.525000
O	1.683000	0.279000	3.787000

CuO24

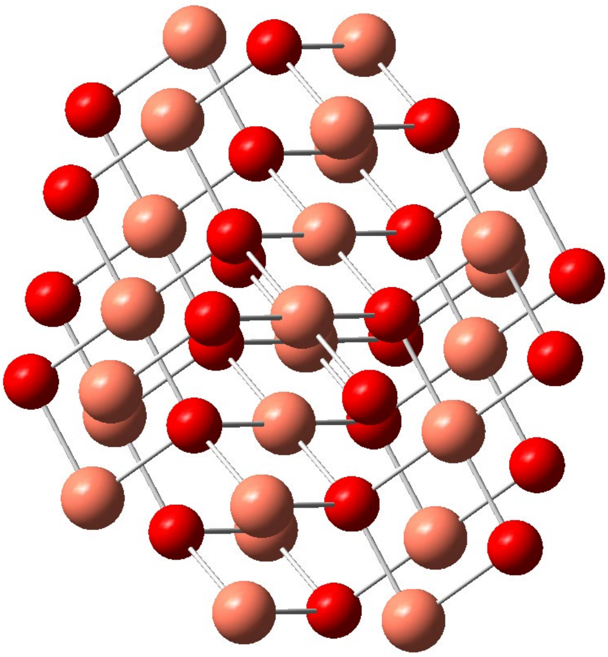

Figure S3. CuO24.

48

Cu	-3.072000	-0.855000	-2.525000
O	-1.683000	-0.279000	-3.787000
Cu	-3.510000	-2.566000	0.000000
Cu	-3.947000	-0.855000	2.525000
O	-2.559000	-3.700000	1.263000
O	-2.559000	-0.279000	1.263000
O	-2.121000	-3.141000	-1.263000
Cu	-3.072000	2.566000	-2.525000
Cu	-3.510000	0.855000	0.000000
O	-2.559000	3.141000	1.263000
O	-2.121000	0.279000	-1.263000
O	0.656000	-1.989000	-3.787000
Cu	-0.732000	-2.566000	-2.525000
Cu	1.607000	-0.855000	-2.525000
O	2.996000	-0.279000	-3.787000
Cu	1.170000	-2.566000	0.000000
O	-0.219000	-1.989000	1.263000
Cu	-1.607000	-2.566000	2.525000
Cu	-1.170000	-4.276000	0.000000
Cu	-1.170000	-0.855000	0.000000
Cu	0.732000	-0.855000	2.525000
O	0.219000	-1.431000	-1.263000
O	2.121000	-3.700000	1.263000
O	2.121000	-0.279000	1.263000
O	2.559000	-3.141000	-1.263000
O	0.656000	1.431000	-3.787000
Cu	-0.732000	0.855000	-2.525000
Cu	1.607000	2.566000	-2.525000
Cu	1.170000	0.855000	0.000000
O	-0.219000	1.431000	1.263000
Cu	-1.607000	0.855000	2.525000
Cu	-1.170000	2.566000	0.000000
Cu	0.732000	2.566000	2.525000
O	0.219000	1.989000	-1.263000
O	2.121000	3.141000	1.263000

O	2.559000	0.279000	-1.263000
O	-0.656000	-1.431000	3.787000
O	-0.656000	1.989000	3.787000
O	-2.996000	0.279000	3.787000
Cu	3.072000	-2.566000	2.525000
Cu	3.510000	-0.855000	0.000000
Cu	3.947000	0.855000	-2.525000
Cu	3.072000	0.855000	2.525000
Cu	3.510000	2.566000	0.000000
O	1.683000	0.279000	3.787000
Cu	1.170000	4.276000	0.000000
O	-2.121000	3.700000	-1.263000
O	2.559000	3.700000	-1.263000

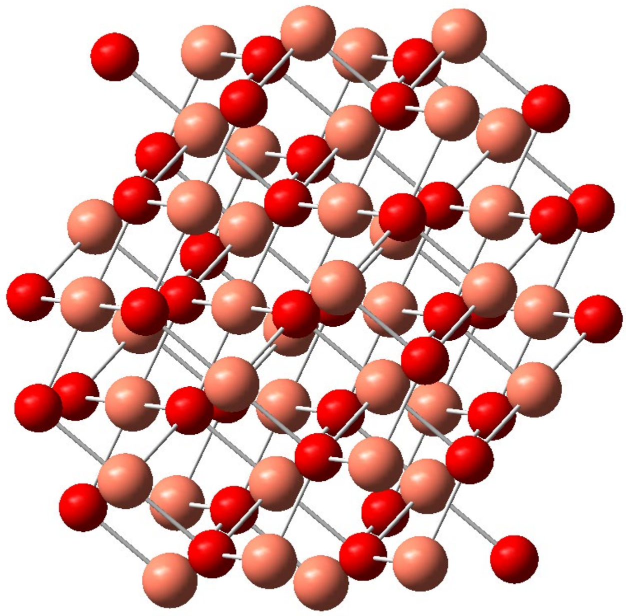

CuO38

Figure S4. CuO38.

76

Cu	-3.072000	-0.855000	-2.525000
O	-1.683000	-3.700000	-3.787000
O	-1.683000	-0.279000	-3.787000
Cu	-3.510000	-2.566000	0.000000
O	-4.898000	-1.989000	1.263000
Cu	-3.947000	-0.855000	2.525000
O	-4.461000	-1.431000	-1.263000
O	-2.559000	-3.700000	1.263000
O	-2.559000	-0.279000	1.263000
O	-2.121000	-3.141000	-1.263000
Cu	-3.072000	2.566000	-2.525000
O	-1.683000	3.141000	-3.787000
Cu	-3.510000	0.855000	0.000000
O	-4.898000	1.431000	1.263000
Cu	-3.947000	2.566000	2.525000
O	-4.461000	1.989000	-1.263000
O	-2.559000	3.141000	1.263000
O	-2.121000	0.279000	-1.263000
O	0.656000	-1.989000	-3.787000
Cu	-0.732000	-2.566000	-2.525000
Cu	-0.295000	-0.855000	-5.050000
Cu	1.607000	-4.276000	-2.525000
Cu	1.607000	-0.855000	-2.525000
O	2.996000	-0.279000	-3.787000
Cu	1.170000	-2.566000	0.000000
O	-0.219000	-1.989000	1.263000
Cu	-1.607000	-2.566000	2.525000
Cu	-1.170000	-4.276000	0.000000
Cu	-1.170000	-0.855000	0.000000
Cu	0.732000	-4.276000	2.525000
Cu	0.732000	-0.855000	2.525000
O	0.219000	-4.852000	-1.263000
O	0.219000	-1.431000	-1.263000
O	2.121000	-3.700000	1.263000
O	2.121000	-0.279000	1.263000

O	2.559000	-3.141000	-1.263000
Cu	2.045000	0.855000	-5.050000
O	0.656000	1.431000	-3.787000
Cu	-0.732000	0.855000	-2.525000
Cu	-0.295000	2.566000	-5.050000
Cu	1.607000	2.566000	-2.525000
Cu	1.170000	0.855000	-0.000000
O	-0.219000	1.431000	1.263000
Cu	-1.607000	0.855000	2.525000
Cu	-1.170000	2.566000	0.000000
Cu	0.732000	2.566000	2.525000
O	0.219000	1.989000	-1.263000
O	2.121000	3.141000	1.263000
O	2.559000	0.279000	-1.263000
O	-0.656000	-1.431000	3.787000
O	-0.656000	1.989000	3.787000
O	-2.996000	0.279000	3.787000
Cu	3.947000	-2.566000	-2.525000
O	4.898000	-1.431000	-1.263000
Cu	3.072000	-2.566000	2.525000
O	4.461000	-1.989000	1.263000
Cu	3.510000	-4.276000	0.000000
Cu	3.510000	-0.855000	0.000000
O	1.683000	-3.141000	3.787000
Cu	3.947000	0.855000	-2.525000
O	4.898000	1.989000	-1.263000
Cu	3.072000	0.855000	2.525000
O	4.461000	1.431000	1.263000
Cu	3.510000	2.566000	-0.000000
O	1.683000	0.279000	3.787000
Cu	-2.045000	-0.855000	5.050000
Cu	0.295000	0.855000	5.050000
Cu	0.295000	-2.566000	5.050000
Cu	1.170000	4.276000	-0.000000
Cu	-0.732000	4.276000	-2.525000
O	-0.219000	4.852000	1.263000
O	-2.121000	3.700000	-1.263000
Cu	-1.607000	4.276000	2.525000
O	2.559000	3.700000	-1.263000
O	1.683000	3.700000	3.787000
Cu	-3.510000	4.276000	0.000000

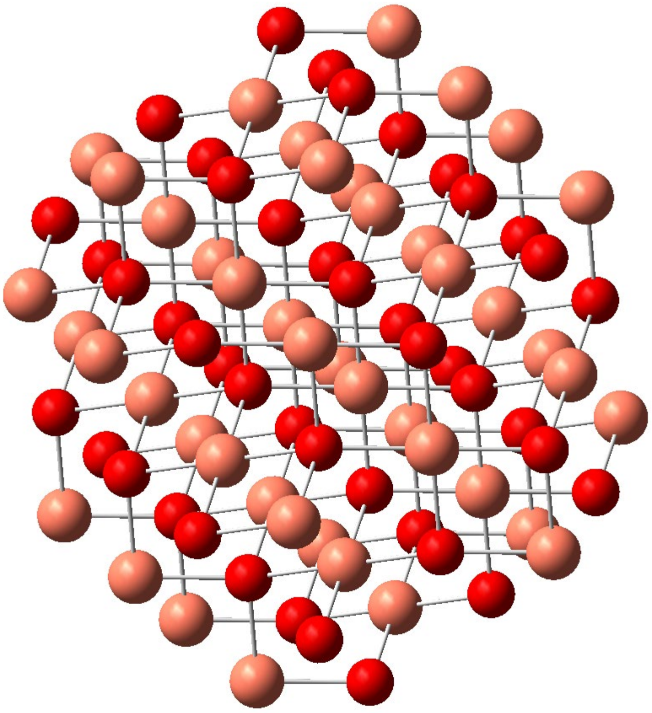

CuO44

Figure S5.

CuO44.
88

O	-4.023000	-1.989000	-3.787000
Cu	-3.072000	-4.276000	-2.525000
Cu	-3.072000	-0.855000	-2.525000
O	-1.683000	-3.700000	-3.787000
O	-1.683000	-0.279000	-3.787000
Cu	-3.510000	-2.566000	0.000000
O	-4.898000	-1.989000	1.263000
Cu	-5.849000	-0.855000	0.000000
Cu	-3.947000	-0.855000	2.525000
O	-4.461000	-1.431000	-1.263000
O	-2.559000	-3.700000	1.263000
O	-2.559000	-0.279000	1.263000
O	-2.121000	-3.141000	-1.263000
Cu	-2.635000	0.855000	-5.050000
O	-4.023000	1.431000	-3.787000
Cu	-3.072000	2.566000	-2.525000
O	-1.683000	3.141000	-3.787000
Cu	-3.510000	0.855000	0.000000
O	-4.898000	1.431000	1.263000
Cu	-3.947000	2.566000	2.525000
O	-4.461000	1.989000	-1.263000
O	-2.559000	3.141000	1.263000
O	-2.121000	0.279000	-1.263000
O	0.656000	-1.989000	-3.787000
Cu	-0.732000	-2.566000	-2.525000
Cu	-0.295000	-0.855000	-5.050000
Cu	1.607000	-4.276000	-2.525000
Cu	1.607000	-0.855000	-2.525000
O	2.996000	-0.279000	-3.787000
Cu	1.170000	-2.566000	0.000000
O	-0.219000	-1.989000	1.263000
Cu	-1.607000	-2.566000	2.525000

Cu	-1.170000	-4.276000	0.000000
Cu	-1.170000	-0.855000	0.000000
Cu	0.732000	-4.276000	2.525000
Cu	0.732000	-0.855000	2.525000
O	0.219000	-4.852000	-1.263000
O	0.219000	-1.431000	-1.263000
O	2.121000	-3.700000	1.263000
O	2.121000	-0.279000	1.263000
O	2.559000	-3.141000	-1.263000
Cu	2.045000	0.855000	-5.050000
O	0.656000	1.431000	-3.787000
Cu	-0.732000	0.855000	-2.525000
Cu	-0.295000	2.566000	-5.050000
Cu	1.607000	2.566000	-2.525000
O	2.996000	3.141000	-3.787000
Cu	1.170000	0.855000	-0.000000
O	-0.219000	1.431000	1.263000
Cu	-1.607000	0.855000	2.525000
Cu	-1.170000	2.566000	0.000000
Cu	0.732000	2.566000	2.525000
O	0.219000	1.989000	-1.263000
O	2.121000	3.141000	1.263000
O	2.559000	0.279000	-1.263000
O	-0.656000	-1.431000	3.787000
O	-2.996000	-3.141000	3.787000
O	-0.656000	1.989000	3.787000
O	-2.996000	0.279000	3.787000
Cu	3.947000	-2.566000	-2.525000
O	4.898000	-1.431000	-1.263000
Cu	3.072000	-2.566000	2.525000
O	4.461000	-1.989000	1.263000
Cu	3.510000	-4.276000	0.000000
Cu	3.510000	-0.855000	0.000000
O	4.023000	-1.431000	3.787000
O	1.683000	-3.141000	3.787000
Cu	3.947000	0.855000	-2.525000
O	4.898000	1.989000	-1.263000
Cu	3.072000	0.855000	2.525000
O	4.461000	1.431000	1.263000
Cu	5.849000	0.855000	-0.000000
Cu	3.510000	2.566000	-0.000000
O	4.023000	1.989000	3.787000
O	1.683000	0.279000	3.787000
Cu	-2.045000	-0.855000	5.050000
Cu	0.295000	0.855000	5.050000
Cu	0.295000	-2.566000	5.050000
Cu	1.170000	4.276000	-0.000000
Cu	-0.732000	4.276000	-2.525000
O	-0.219000	4.852000	1.263000
O	-2.121000	3.700000	-1.263000
Cu	-1.607000	4.276000	2.525000
Cu	2.635000	-0.855000	5.050000
O	2.559000	3.700000	-1.263000
Cu	3.072000	4.276000	2.525000
O	1.683000	3.700000	3.787000
Cu	-3.510000	4.276000	0.000000

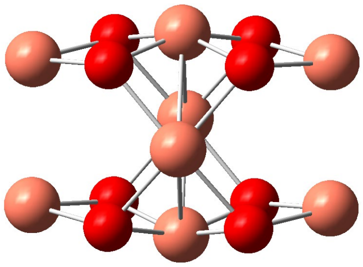

CuO8_opt

Figure S6. CuO8_opt.

```
16

 O     -2.070325      0.023723      0.977794
 O     -1.828127      0.158858     -1.369712
Cu      1.089919     -0.774498     -2.456223
Cu      1.152939     -1.271038      0.045569
 O     -0.214558     -2.022923      1.051596
Cu     -1.220602     -1.113735     -0.190175
Cu      0.579195     -1.065790      2.521713
 O      0.028249     -1.886881     -1.296189
 O      1.828127     -0.158858      1.369712
Cu     -0.579195      1.065790     -2.521713
Cu      1.220602      1.113735      0.190175
 O     -0.028249      1.886881      1.296189
Cu     -1.089919      0.774498      2.456223
Cu     -1.152939      1.271038     -0.045569
 O      0.214558      2.022923     -1.051596
 O      2.070325     -0.023723     -0.977794
```

CuO16_opt

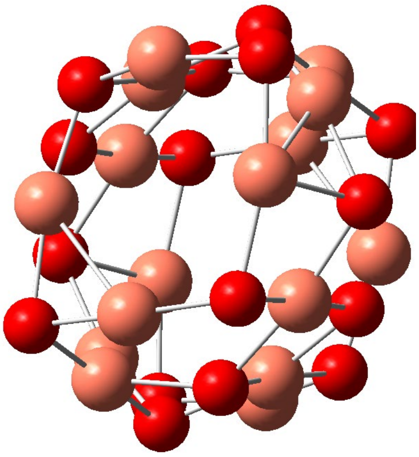

Figure S7. CuO16_opt.

32

Cu	-0.766807	-0.772067	-4.102777
O	-2.210636	-0.386214	-2.919703
O	-2.641448	-1.415678	1.951641
O	-0.785437	-2.506608	-3.928865
Cu	-2.420513	0.363514	1.616430
O	-1.851898	2.093771	1.542204
O	-2.091281	1.175635	-0.774700
Cu	-1.546452	-1.885132	-2.131756
Cu	0.776898	-2.476612	-2.967768
Cu	0.861793	-2.169578	-0.049025
O	0.117824	-2.316753	1.637836
Cu	-1.215742	-1.246969	3.035662
Cu	-1.588438	-2.195031	0.757494
Cu	1.264664	-1.052284	2.348492
O	-0.745362	-2.839909	-0.725995
O	2.091281	-1.175635	0.774700
O	1.851898	-2.093771	-1.542204
O	-0.365283	1.023227	-3.952834
Cu	-1.264664	1.052284	-2.348492
Cu	1.215742	1.246969	-3.035662
Cu	1.588438	2.195031	-0.757494
O	0.745362	2.839909	0.725995
Cu	-0.776898	2.476612	2.967768
Cu	-0.861793	2.169578	0.049025
Cu	1.546452	1.885132	2.131756
O	-0.117824	2.316753	-1.637836
O	0.785437	2.506608	3.928865
O	2.641448	1.415678	-1.951641
O	0.365283	-1.023227	3.952834
Cu	2.420513	-0.363514	-1.616430
Cu	0.766807	0.772067	4.102777
O	2.210636	0.386214	2.919703

CuO24_opt

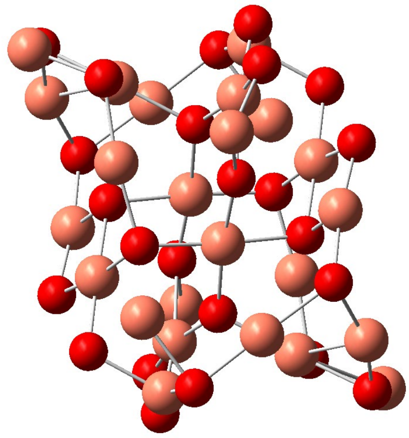

Figure S8. CuO24_opt.

48

Cu	-2.940874	-1.658160	-1.988669
O	-1.383110	-1.007322	-3.558877
Cu	-2.166558	-3.416696	-0.278017
Cu	-3.902055	-0.032300	2.084671
O	-1.969354	-3.469885	1.600694
O	-2.168922	-0.370260	1.325303
O	-2.158445	-3.358186	-2.125398
Cu	-2.849487	4.551261	-0.428143
Cu	-2.648756	1.449657	-0.453647
O	-2.863006	3.006074	0.463874
O	-2.021347	-0.115228	-1.223549
O	0.923431	-2.396007	-3.455049
Cu	-0.704219	-2.583051	-2.895546
Cu	1.608077	-1.162940	-2.357774
O	3.030905	-0.954612	-3.356751
Cu	0.942312	-3.316767	-0.360892
O	0.081983	-1.648608	1.458131
Cu	-1.265168	-2.119045	2.566865
Cu	-0.483320	-4.479198	1.208218
Cu	-0.985059	-0.940228	0.086920
Cu	0.800899	-0.318062	2.523306
O	0.277546	-1.693658	-1.079133
O	1.096355	-4.957340	0.438825
O	2.021347	0.115228	1.223549
O	2.863006	-3.006074	-0.463874
O	0.469203	0.965575	-3.608276
Cu	-0.800899	0.318062	-2.523306
Cu	1.265168	2.119045	-2.566865
Cu	0.985059	0.940228	-0.086920
O	-0.277546	1.693658	1.079133
Cu	-1.608077	1.162940	2.357774
Cu	-0.942312	3.316767	0.360892
Cu	0.704219	2.583051	2.895546
O	-0.081983	1.648608	-1.458131
O	2.158445	3.358186	2.125398
O	2.168922	0.370260	-1.325303
O	-0.469203	-0.965575	3.608276
O	-0.923431	2.396007	3.455049
O	-3.030905	0.954612	3.356751
Cu	2.849487	-4.551261	0.428143
Cu	2.648756	-1.449657	0.453647
Cu	3.902055	0.032300	-2.084671

Cu	2.940874	1.658160	1.988669
Cu	2.166558	3.416696	0.278017
O	1.383110	1.007322	3.558877
Cu	0.483320	4.479198	-1.208218
O	-1.096355	4.957340	-0.438825
O	1.969354	3.469885	-1.600694

Figure S9 – Theoretical infrared spectrum calculated with DFT for CuO8 and CuO16 relaxed models

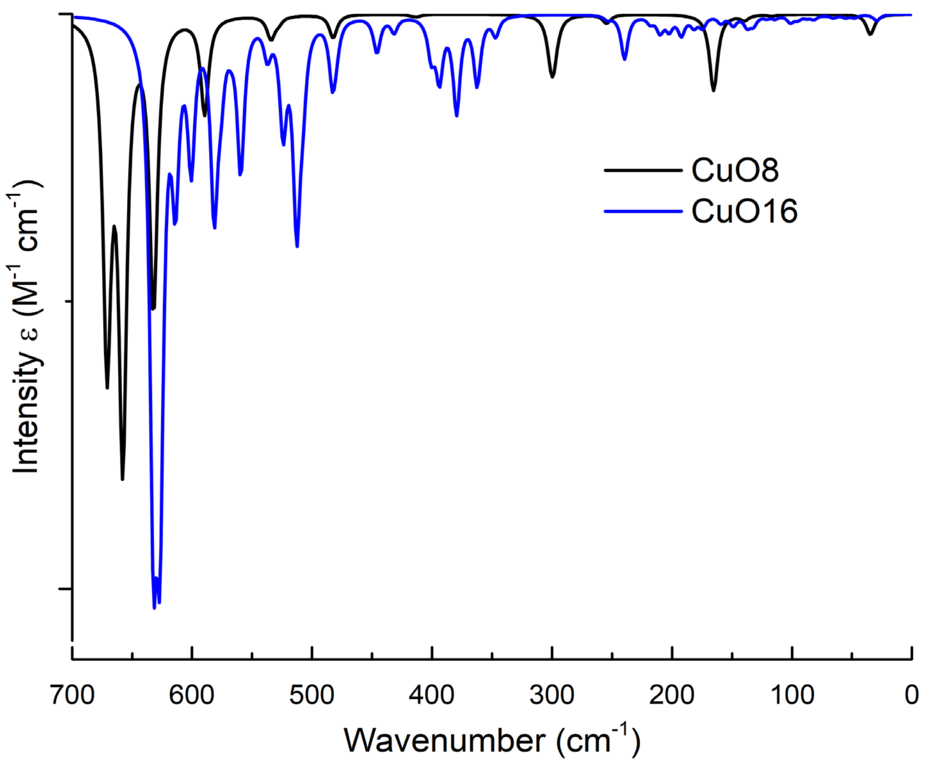

Supplement: Supplementary file 1 [file materials-16-04800-s001.zip › materials-2451714-supplementary.pdf]
